# Error Management Training and Adaptive Expertise in Learning Computed Tomography Interpretation: A Randomized Clinical Trial

**DOI:** 10.1001/jamanetworkopen.2024.31600

**Published:** 2024-09-09

**Authors:** Leonardo Aliaga, Rebecca A. Bavolek, Benjamin Cooper, Amy Mariorenzi, James Ahn, Aaron Kraut, David Duong, Catherine Burger, Michael A. Gisondi

**Affiliations:** 1 Department of Emergency Medicine, Stanford University, Stanford, California; 2 Department of Emergency Medicine, University of California, Los Angeles; 3 Department of Emergency Medicine, University of Texas Health Science Center at Houston; 4 Department of Emergency Medicine, Brown University, Providence, Rhode Island; 5 Division of the Biological Sciences, University of Chicago, Chicago, Illinois; 6 Department of Emergency Medicine, University of Wisconsin School of Medicine and Public Health, Madison; 7 Department of Emergency Medicine, Highland Hospital, Alameda Health System, Oakland, California; 8 Department of Emergency Medicine, Vanderbilt University Medical Center, Nashville, Tennessee

## Abstract

**Importance:**

Adaptive expertise helps physicians apply their skills to novel clinical cases and reduce preventable errors. Error management training (EMT) has been shown to improve adaptive expertise with procedural skills; however, its application to cognitive skills in medical education is unclear.

**Objective:**

To evaluate whether EMT improves adaptive expertise when learning the cognitive skill of head computed tomography (CT) interpretation.

**Design, Setting, and Participants:**

This 3-arm randomized clinical trial was conducted from July 8, 2022, to March 30, 2023, in 7 geographically diverse emergency medicine residency programs. Participants were postgraduate year 1 through 4 emergency medicine residents masked to the hypothesis.

**Interventions:**

Participants were randomized 1:1:1 to a difficult EMT, easy EMT, or error avoidance training (EAT) control learning strategy for completing an online head CT curriculum. Both EMT cohorts received no didactic instruction before scrolling through head CT cases, whereas the EAT group did. The difficult EMT cohort answered difficult questions about the teaching cases, leading to errors, whereas the easy EMT cohort answered easy questions, leading to fewer errors. All 3 cohorts used the same cases.

**Main Outcomes and Measures:**

The primary outcome was a difference in adaptive expertise among the 3 cohorts, as measured using a head CT posttest. Secondary outcomes were (1) differences in routine expertise, (2) whether the quantity of errors during training mediated differences in adaptive expertise, and (3) the interaction between prior residency training and the learning strategies.

**Results:**

Among 212 randomized participants (mean [SD] age, 28.8 [2.0] years; 107 men [50.5%]), 70 were allocated to the difficult EMT, 71 to the easy EMT, and 71 to the EAT control cohorts; 150 participants (70.8%) completed the posttest. The difficult EMT cohort outperformed both the easy EMT and EAT cohorts on adaptive expertise cases (60.6% [95% CI, 56.1%-65.1%] vs 45.2% [95% CI, 39.9%-50.6%], vs 40.9% [95% CI, 36.0%-45.7%], respectively; *P* < .001), with a large effect size (η^2^ = 0.19). There was no significant difference in routine expertise. The difficult EMT cohort made more errors during training than the easy EMT cohort. Mediation analysis showed that the number of errors during training explained 87.2% of the difficult EMT learning strategy’s effect on improving adaptive expertise (*P* = .01). The difficult EMT learning strategy was more effective in improving adaptive expertise for residents earlier in training, with a large effect size (η^2^ = 0.25; *P* = .002).

**Conclusions and Relevance:**

In this randomized clinical trial, the findings show that EMT is an effective method to develop physicians’ adaptive expertise with cognitive skills.

**Trial Registration:**

ClinicalTrials.gov Identifier: NCT05284838

## Key Points

**Question:**

Does error management training, characterized by guiding learners to make errors during training, improve adaptive expertise when used to teach a cognitive skill in medical education?

**Findings:**

In this randomized clinical trial of 150 emergency medicine residents, making more errors while learning head computed tomography interpretation significantly decreased diagnostic errors when tested with new head computed tomography cases compared with active learning with few errors and a passive learning control.

**Meaning:**

These findings suggest that error management training improves adaptive expertise, which may help reduce future errors when physicians face new or unfamiliar cases.

## Teaser

This randomized clinical trial evaluates the effectiveness of error management training in cognitive learning of head computed tomography interpretation among emergency medicine residents.

## Introduction

Reducing future medical errors is a cornerstone of medical education, yet physicians still make errors when facing unfamiliar clinical cases.^1,2^ Adaptive expertise is the ability to apply existing knowledge and skills to solve novel problems and manage unfamiliar situations.^3,4,5,6^ Adaptive expertise may help physicians reduce future errors when managing unfamiliar clinical cases.^2,7,8^ However, adaptive expertise is difficult to teach, and residency curricula often do not emphasize or assess this skill.^6^

Error management training (EMT) is a teaching method that may develop physicians’ adaptive expertise.^9,10,11^ In EMT, learners are asked to solve difficult problems before they are taught how to solve them. Importantly, errors are encouraged while learning. These errors focus learners’ attention on a problem’s underlying conceptual features, which improves their ability to apply learned skills to new problems (ie, develop adaptive expertise).^3,7,9,12,13^ Experimental studies in medical education have used EMT to improve adaptive expertise in procedural skills such as ultrasonography, central venous catheterization, and fasciotomy.^14,15,16^ However, EMT’s application to developing adaptive expertise with cognitive skills in medical education is underexplored. We aimed to fill this gap, given that physicians use cognitive skills across every domain of practice.

Making errors and active learning (active exploration or experimentation with a skill before receiving didactic instruction) are both components of EMT.^9,11^ However, it is unclear whether improved learning outcomes in prior EMT studies were due to making errors, active learning, or both.^14,15^ These EMT studies used error avoidance training (EAT) as a control condition, where learners first passively receive instruction on a skill (passive learning) and are told to avoid errors. To date, there have been no studies that separately controlled for the 2 components of EMT (making errors and active learning). Distinguishing between the these components may guide the use of EMT in medical education curricula.

We aimed to determine whether EMT improves adaptive expertise for a cognitive skill in medical education, using focused head computed tomography (CT) interpretation as a model. Identifying time-sensitive head CT pathology is a key skill for emergency physicians that is not well covered in residency training.^17^ Diagnosing intracranial hemorrhage and increased intracranial pressure at the time of scanning may expedite neurosurgery consultation and critical interventions.^18,19^ Emergency physicians may improve patient care by rapidly identifying these specific abnormalities. We used a population of emergency medicine residents, who are familiar with head CT interpretation but lack proficiency. Head CT scans provide the wide case variation needed to test residents’ adaptive expertise. We hypothesized that EMT, compared with EAT, would improve adaptive expertise when used to teach head CT interpretation to emergency medicine residents. We also aimed to determine whether the experience of making errors, separate from active learning, is needed to improve adaptive expertise through EMT, hypothesizing that the number of errors made during training would positively mediate improvement in adaptive expertise.

## Methods

This multicenter, 3-arm, active-controlled, parallel-group randomized clinical trial was conducted from July 8, 2022, to March 30, 2023. The Stanford University Institutional Review Board approved this study. All participants gave written informed consent. This study followed the Consolidated Standards of Reporting Trials Extension (CONSORT Extension) reporting guideline.^20^ The trial protocol is available in Supplement 1.

### Participants

We recruited emergency medicine residents in their postgraduate years (PGYs) 1 through 4 from 7 geographically diverse residency programs. We included PGY 1 through 3 and PGY 1 through 4 programs to strengthen generalizability. Data on race and ethnicity were not collected as this was not clinically or scientifically relevant to the research question. Participants who completed the intervention and posttest received a $10 gift card. Participants were masked to their cohort allocation, the details of each learning strategy, and the study hypotheses.

### Randomization

**Figure 1.**
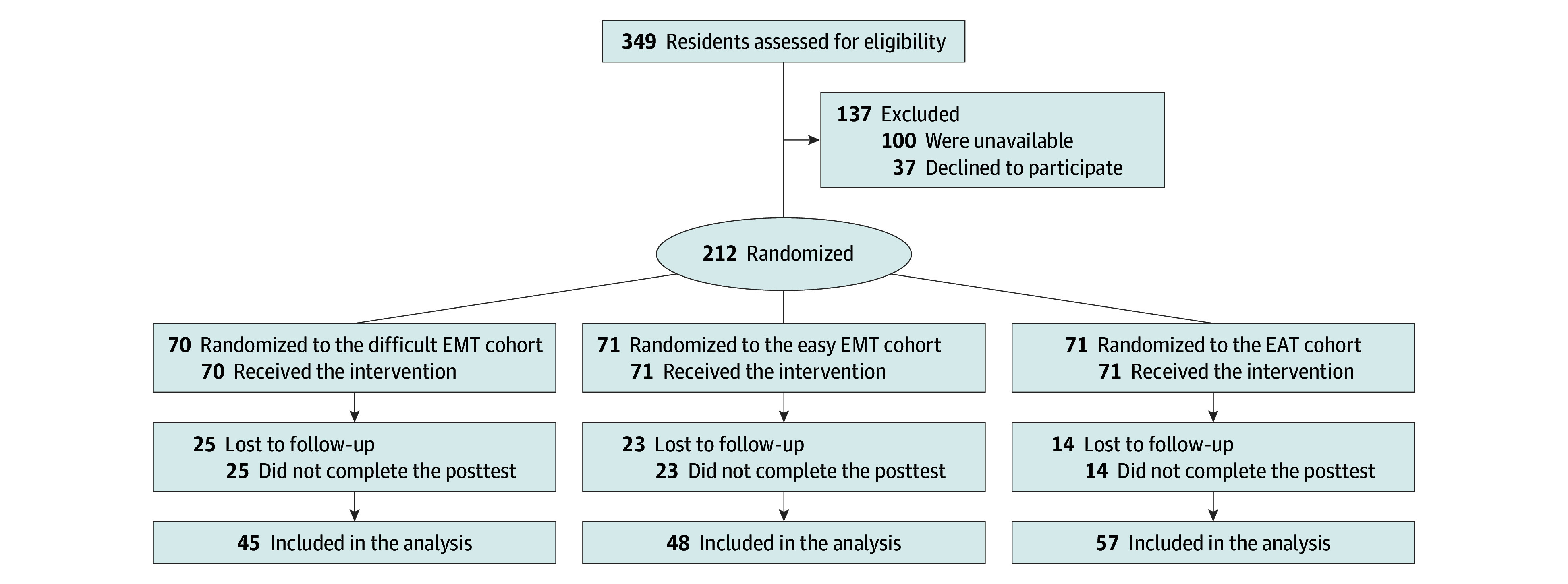
Participant Flowchart EAT indicates error avoidance training; EMT, error management training.

Author L.A. stratified participants by PGY within a site and then randomized them to 1 of 3 cohorts (1:1:1) using permuted block randomization (Figure 1): difficult EMT, easy EMT, and EAT control. We generated block randomization lists for each site using Research Randomizer.^21^ Each residency program was individually randomized using this same method. Within-site randomization allowed prior program-specific radiology education to be distributed among the 3 cohorts. All programs’ prior radiology education was randomly interspersed throughout their didactic curriculum, and no program had a formal radiology rotation.

### Study Procedure

Participants individually completed a 1-hour online curriculum on head CT interpretation immediately followed by a 1-hour posttest. We administered the curriculum and posttest during each residency program’s weekly didactic conference. We emailed participants a link to access the curriculum on 1 of 3 websites created for the study, based on their assigned cohort. At the beginning of the second hour, we emailed participants a link to the posttest (links to websites and posttest available in the eMethods in Supplement 2).

We did not use a pretest to assess baseline skills. Pretests can skew study results by altering how participants learn during the intervention.^22^ Randomization should equalize differences in baseline skill, and a posttest-only design is recommended in education research when each cohort has at least 40 participants.^22^

### Online Radiology Platform

We used Pacsbin (Orion Medical Technologies) to host the teaching and testing head CT cases. Pacsbin is a web-based radiology picture archiving and communication system (PACS) that allows users to scroll through CT scans just as they would on a radiology viewer in the emergency department.^23^ Viewing radiology cases on a PACS reproduces the cognitive work involved in head CT interpretation, which was important for making our results generalizable to clinical training and practice. Another study previously showed the feasibility of using Pacsbin for emergency radiology education.^24^

### Interventions

The interventions were the difficult EMT and easy EMT learning strategies. We adapted a head CT curriculum from a study involving emergency medicine residents.^24^ Our study’s curriculum had 9 head CT cases (content on intracranial hemorrhage and increased intracranial pressure). All 3 cohorts used the same teaching cases, received the same written educational content, and could scroll through head CTs on Pacsbin. The learning strategy used to present the content differed among cohorts (experimental design shown in the eFigure in Supplement 2).

#### Intervention Cohorts

For each teaching case, the difficult EMT and easy EMT cohorts first scrolled through a head CT case to identify critical findings. Then, the difficult EMT cohort answered difficult questions about that teaching case, which were expected to lead to errors. In contrast, the easy EMT cohort answered easy questions, which were expected to lead to fewer errors. After answering the questions, both cohorts reviewed an explanations webpage with didactic teaching content on that case. Modulating the question difficulty for the 2 EMT cohorts was the essential experimental manipulation that allowed us to separate the effects of making errors from active learning. Question difficulty varied through the specificity of knowledge being asked. For example, the difficult EMT cohort had to identify specific types of intracranial hemorrhage and write where they saw them. The easy EMT cohort only had to identify any type of intracranial hemorrhage. Question difficulty was tested and revised using pilot residents. The easy EMT cohort primarily used active learning and served as a control for the errors made by the difficult EMT cohort.

#### Control Cohort

The EAT cohort received only the explanations page for each teaching case. Participants could scroll through the CT case but were first shown how to identify the critical findings. The EAT cohort used passive learning (passively received information without being challenged beyond that didactic instruction) and served as a control for active learning.

### Head CT Posttest

We adapted a head CT interpretation test from a study that assessed diagnostic accuracy using Pacsbin. The study collected content and response process validity evidence for the test.^24^ No other published instrument uses a radiology PACS to assess head CT diagnostic accuracy in emergency medicine residents. Other studies used head CT interpretation tests with static images or basic knowledge questions, limiting transferability to clinical practice.^25,26,27^

We collected additional validity evidence for using the head CT test in this study. We piloted the adapted test in 12 PGYs 1 through 4 emergency medicine residents, using 3 residents per PGY level. Residents who participated in the pilot phase were not included in the final study cohort. We used feedback from the pilot residents to adjust question wording, case difficulty, and test length. We conducted an item analysis for test questions using their discrimination index and item difficulty. Test questions with a discrimination index less than 0.2 and mean score less than 30% (high difficulty) were revised or removed.

The posttest included 23 questions and 18 unique head CT cases, with a mix of novel cases to assess adaptive expertise and familiar cases to assess routine expertise. The adaptive expertise cases required participants to transfer learned concepts to novel cases that were dissimilar from what they saw in the training cases. In contrast, the routine expertise cases more closely mimicked the training cases.

### Outcome Measures

Our primary outcome was adaptive expertise, as measured by posttest scores on the novel cases. There were 3 secondary outcomes. First, we measured routine expertise through posttest scores on the familiar cases. Second, we assessed whether the number of errors mediated differences in adaptive expertise between the 2 EMT cohorts. We measured the number of errors both EMT cohorts made on the training cases. We then conducted a mediation analysis, with learning strategy as the exposure variable, number of errors during training as the mediator variable, and score on adaptive expertise cases as the outcome variable. Third, we measured the interaction effect between prior residency training experience and the learning strategies (using PGY level as a proxy). We stratified posttest scores in all cohorts by PGY level to examine interaction effects. Both PGY 3 and PGY 4 participants were grouped together as senior residents since there was an insufficient number of PGY 4 participants for their own comparison group.

### Statistical Analysis

Our power calculation showed that a total of 138 participants was required to detect a 6% difference (medium effect size) between the control cohort and either intervention cohort with 80% power (α = .05). We used the standard deviation of the head CT test from a previous study for our power calculation.^24^

We compared participant baseline characteristics by cohort using Kruskal-Wallis tests for continuous covariates or χ^2^ tests for categorical covariates. We also compared differences in baseline characteristics between participants who completed the posttest and those who did not.

We compared posttest scores on the adaptive and routine expertise cases using 1-way analysis of variance (ANOVA). We performed 3 pairwise planned contrasts for the adaptive expertise cases: difficult EMT vs easy EMT, difficult EMT vs EAT, and easy EMT vs EAT. A Bonferroni adjustment for multiple comparisons set the prespecified α at *P* < .017 for these 3 planned contrasts. We compared the number of errors made on the training cases by the difficult EMT and easy EMT cohorts using a 2-tailed independent samples *t* test. We performed a mediation analysis using the Hayes PROCESS macro for SPSS, version 4.2 (IBM Corporation).^28^ Our causal model assumed that the learning strategy (difficult EMT vs easy EMT) would lead participants to experience errors during training. These errors would then show participants their knowledge gaps and improve their conceptual understanding of head CT interpretation. Participants could then better transfer learned concepts to novel head CT cases. We assessed the interaction between PGY level and learning strategy on posttest scores using 2-way ANOVAs (factors were cohort and PGY level). Post hoc pairwise comparisons were performed using Tukey test, which accounts for multiple comparisons.

We calculated effect sizes using η^2^ and Cohen *d*, where applicable. We performed all statistical analyses using SPSS, version 29 software.

## Results

### Demographics

A total of 212 participants were randomized (mean [SD] age, 28.8 [2.0] years; 107 men [50.5%] and 105 women [49.5%]), with 70 in the difficult EMT cohort, 71 in the easy EMT cohort, and 71 in the EAT control cohort. Of those 212 participants, 150 (70.8%) completed the posttest and were included in the analysis, with 45 (30.0%) in the difficult EMT cohort, 48 (32.0%) in the easy EMT cohort, and 57 (38.0%) in the EAT control cohort. The posttest completion rate was 64.3% for the difficult EMT cohort, 67.6% for the easy EMT cohort, and 80.3% for the EAT control cohort. Baseline characteristics of the 150 participants included in the analysis were well balanced across cohorts (Table). There were no significant differences in baseline characteristics or proportion within cohorts between participants who completed the posttest and those who did not (eTable 1 in Supplement 2). Baseline characteristics were also well balanced among all 212 participants initially randomized (eTable 2 in Supplement 2).

**Table.** Characteristics of Participants Included in the Analysis

Characteristic	No. of participants (%)				*P* value^a^
	Overall (n = 150)	Difficult EMT (n = 45)	Easy EMT (n = 48)	EAT control (n = 57)	
Age, mean (SD), y	28.9 (2.1)	28.8 (2.3)	29.0 (2.5)	29.0 (1.6)	.17
Sex					
Female	74 (49.3)	21 (46.7)	26 (54.2)	27 (47.4)	.72
Male	76 (50.7)	24 (53.3)	22 (45.8)	30 (52.6)	
Postgraduate year					
1	45 (30.0)	15 (33.3)	16 (33.3)	14 (24.6)	.65
2	48 (32.0)	14 (31.1)	15 (31.3)	19 (33.3)	
3	44 (29.3)	14 (31.1)	14 (29.2)	16 (28.1)	
4	13 (8.7)	2 (4.4)	3 (6.3)	8 (14.0)	
Institution					
University of California, Los Angeles	28 (18.7)	9 (20.0)	6 (12.5)	13 (22.8)	.80
Highland Hospital	16 (10.7)	5 (11.1)	7 (14.6)	4 (7.0)	
Brown University	19 (12.7)	5 (11.1)	5 (10.4)	9 (15.8)	
University of Chicago	15 (10.0)	6 (13.3)	5 (10.4)	4 (7.0)	
University of Wisconsin	18 (12.0)	6 (13.3)	5 (10.4)	7 (12.3)	
University of Texas Health Science Center at Houston	36 (24.0)	7 (15.6)	14 (29.2)	15 (26.3)	
Vanderbilt University	18 (12.0)	7 (15.6)	6 (12.5)	5 (8.8)	
Program length					
4 y	63 (42.0)	19 (42.2)	18 (37.5)	26 (45.6)	.70
3 y	87 (58.0)	26 (57.8)	30 (62.5)	31 (54.4)	

Abbreviations: EAT, error avoidance training; EMT, error management training.

^a^ Kruskal-Wallis test or Pearson χ^2^ test.

### Adaptive Expertise

**Figure 2.**
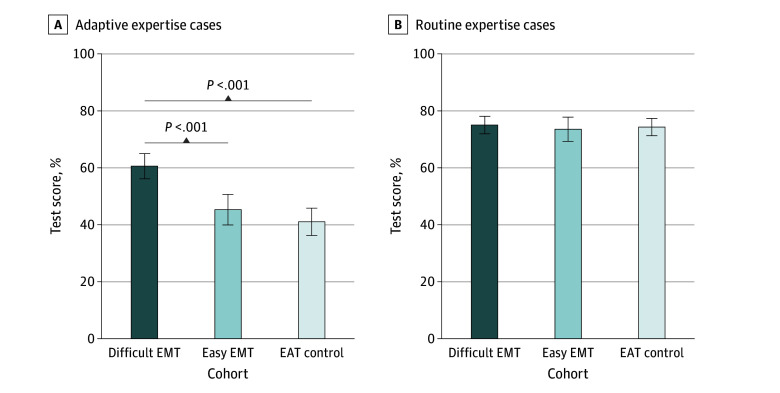
Posttest Performance on Adaptive Expertise and Routine Expertise Cases Significant pairwise comparisons were planned contrasts. Error bars indicate 95% CI. EAT indicates error avoidance training; EMT, error management training.

For the primary outcome, the difficult EMT cohort outperformed both the easy EMT and EAT control cohorts on the novel cases assessing adaptive expertise (Figure 2A). Mean posttest scores on the adaptive expertise cases were 60.6% (95% CI, 56.1%-65.1%) in the difficult EMT cohort, 45.2% (95% CI, 39.9%-50.6%) in the easy EMT cohort, and 40.9% (95% CI, 36.0%-45.7%) in the EAT control cohort. The 1-way ANOVA results were statistically significant, with a large effect size (η^2^ = 0.19; *P* < .001). Planned contrasts showed significant differences between the difficult EMT and easy EMT cohorts, with a medium effect size (η^2^ = 0.11; *P* < .001), and between the difficult EMT and EAT control cohorts, with a large effect size (η^2^ = 0.18; *P* < .001). We found no difference between the easy EMT and EAT control cohorts.

### Secondary Outcomes

#### Routine Expertise

We found no difference in performance on the familiar cases assessing routine expertise (Figure 2B). Mean posttest scores on the routine expertise cases were 75.1% (95% CI, 72.0%-78.2%) in the difficult EMT cohort, 73.6% (95% CI, 69.3%-77.9%) in the easy EMT cohort, and 74.4% (95% CI, 71.3%-77.4%) in the EAT control cohort (*P* = .84).

#### Mediating Effect of Errors on Adaptive Expertise

**Figure 3.**
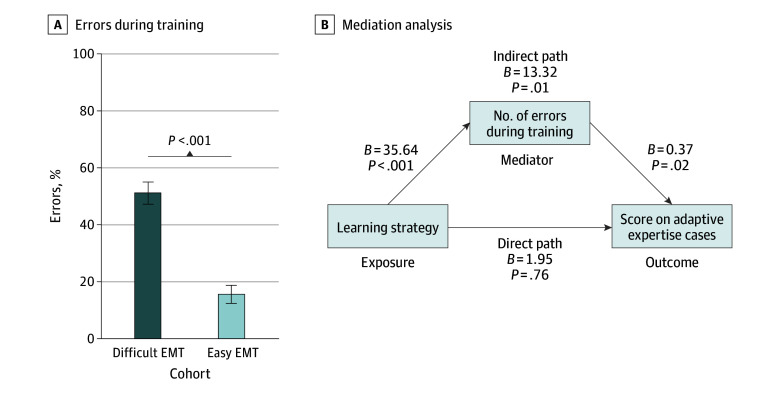
Mediating Effect of Errors on Adaptive Expertise Error bars indicate 95% CIs, and *B* indicates unstandardized β coefficients. EMT indicates error management training.

The number of errors made during training positively mediated improvement in posttest scores on the adaptive expertise cases. The difficult EMT cohort made more errors on the training cases than the easy EMT cohort (51.4% [95% CI, 47.7%-55.2%] vs 15.8% [95% CI, 12.6%-18.9%], respectively; *P* < .001), with a large effect size (Cohen *d* = 3.1) (Figure 3A). Our mediation analysis showed that the learning strategy (difficult EMT vs easy EMT) influenced the number of errors made during training, which in turn influenced posttest scores on the adaptive expertise cases. Taken together, 87.2% of the difficult EMT learning strategy’s effect on improving adaptive expertise was explained by the number of errors made during training (*P* = .01). More errors made during training led to higher posttest scores on the adaptive expertise cases (Figure 3B).

#### Interaction Effect Between PGY Level and Learning Strategy 

**Figure 4.**
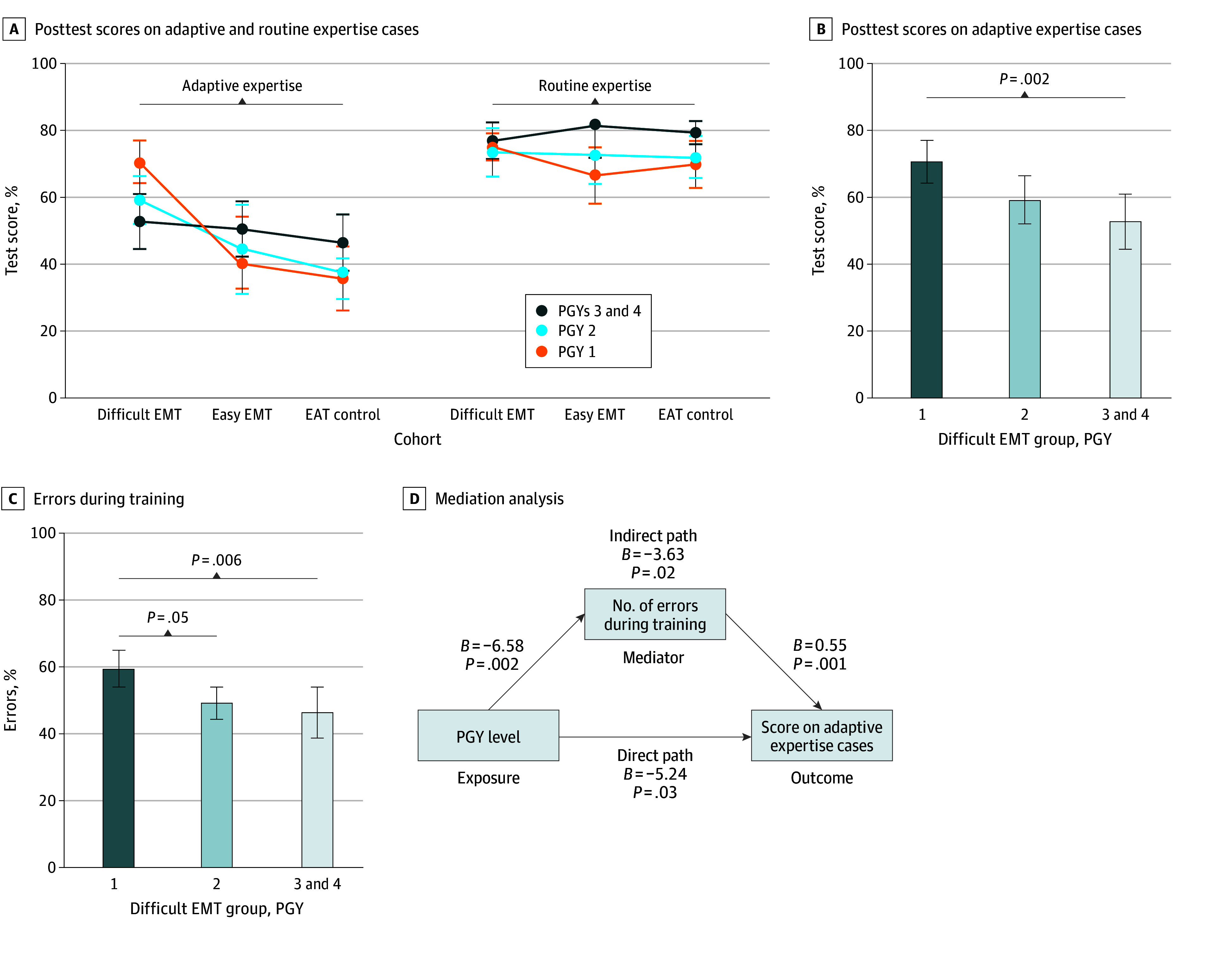
Interaction Effect Between Postgraduate Year (PGY) Level and Learning Strategy A, Posttest scores stratified by PGY level. B, One-way analysis of variance showed a large effect size (η^2^ = 0.25). C, One-way analysis of variance showed a large effect size (η^2^ = 0.21). Post hoc pairwise comparisons in B and C were performed using a Tukey test. D, The direct path remained significant when the mediator variable was included in the multiple regression, indicating partial mediation. Error bars indicate 95% CIs, and *B* indicates unstandardized β coefficients. EAT indicates error avoidance training; EMT, error management training.

Higher PGY level participants performed better in all conditions, except those in the difficult EMT cohort, on the adaptive expertise cases (Figure 4A; eTables 3 and 4 in Supplement 2). Within the difficult EMT cohort, PGY 1 participants scored the highest on the adaptive expertise cases, followed by PGY 2 participants, and then PGY 3 participants and PGY 4 participants (senior residents). The 2-way ANOVA on the adaptive expertise cases showed a significant cohort-PGY interaction, with a medium effect size (η^2^ = 0.10; *P* = .006), indicating that the learning strategies’ effect on improving adaptive expertise depended on the PGY level (eTable 5 in Supplement 2). The difficult EMT learning strategy was more effective in improving adaptive expertise for residents earlier in training, with a large effect size (η^2^ = 0.25; *P* = .002) (Figure 4B). Given this unexpected finding, we examined the error rates by PGY level and found that PGY 1 participants made more errors during training than the participants in higher PGYs, with a large effect size (η^2^ = 0.21; *P* = .006) (Figure 4C). We then conducted a post hoc mediation analysis that showed that 40.9% of the PGY level’s effect on adaptive expertise performance was explained by the number of errors made on the training cases (*P* = .02) (Figure 4D).

The 2-way ANOVA on the routine expertise cases only showed a significant effect of PGY level on posttest scores, with a medium effect size (η^2^ = 0.10; *P* < .001) (eTable 5 in Supplement 1). We conducted sensitivity analyses removing PGY 4 participants from the senior resident factor; the 2-way ANOVA results remained significant (η^2^ = 0.07; *P* = .008) (eTable 6 in Supplement 2).

## Discussion

Adaptive expertise, the ability to transfer learned skills to novel problems, is being increasingly recognized as an important outcome of medical education.^3,6,29,30^ In this multicenter randomized clinical trial, we found that guiding emergency medicine residents to make errors while learning head CT interpretation led to improved adaptive expertise. Residents who made more errors during training made fewer errors when they subsequently evaluated novel head CT cases on a posttest. To our knowledge, this study is the first to use EMT to improve adaptive expertise for a cognitive skill in medical education.

Guiding learners to make errors during learning might appear antithetical to our goal of achieving errorless performance in medical education. However, seeing errors in a negative light overlooks a powerful advantage learners gain by making mistakes. Errors force learners to confront their knowledge gaps and faulty mental schemas.^31,32^ Learners use their wrong answers as contrasting examples to the correct answer, which helps revise their mental schemas.^33,34,35^ That contrast helps learners recognize the conceptual features of a problem, which develops adaptive expertise.^7^ Our study demonstrated the value of making errors during learning as measured by improved adaptive expertise on the posttest. We suspect that residents in the difficult EMT cohort detected gaps in their mental toolbox of reading head CTs because of their errors. Gaining a better understanding of those tools facilitated transfer of their learned skills to the adaptive expertise cases.

Our findings align with the growing literature showing that error-based training methods may improve performance when facing novel or unfamiliar problems.^13,34,36,37,38^ One study, however, found no difference between EMT and EAT among veterinary students learning blood smear analysis.^39^ All participants received didactic instruction before practicing, which may have made EMT more like EAT. All participants could make errors during training and received feedback of correct or incorrect, which may have made EAT more like EMT. The feedback was nonspecific and may not have allowed EMT participants to fully benefit from errors. These differences in approach highlight the importance of allowing struggle before giving instruction and then providing specific feedback after errors.

Interestingly, we found no difference in routine expertise across the 3 learning strategies. Prior studies showed that error-based training methods hindered routine expertise,^11^ which is thought to be from the extra cognitive load that comes with making and processing errors. It may be harder for learners to assimilate basic knowledge, thus compromising routine expertise at the expense of adaptive expertise. It is reassuring that the difficult EMT cohort did no worse on the routine expertise cases than the easy EMT and EAT control cohorts.

We found that the number of errors made during training positively mediated improvement in adaptive expertise. Prior EMT studies noted limitations of being unable to quantify the frequency of errors or their influence on learning outcomes.^14,15,39^ Our study filled this gap by using a design that allowed us to make causal inferences about the effect of errors on adaptive expertise. This insight into EMT’s mechanism of action may inform how we design education curricula. Incorporating difficult problems in didactics and simulation that are just beyond residents’ current abilities may produce the useful types of errors we found in our study. In contrast, passively delivering information with traditional errorless training may hinder residents’ adaptive expertise development.

Interns outperformed senior residents on the adaptive expertise cases within the difficult EMT cohort. This unexpected finding led us to examine a potential mechanism. A participant-level mediation analysis showed that the number of errors made during training positively mediated this improved adaptive expertise. This post hoc mediation analysis followed the same a priori hypothesis for our intervention-level mediation analysis. It may have been easier for interns to lean into their mistakes and learn from them. Another possible interpretation is that the training cases and questions were not difficult enough for the senior residents to trigger enough reflection and growth. There may be an optimal amount of difficulty that could lead to the most adaptive expertise.

It may be tempting to attribute the difficult EMT cohort’s improved adaptive expertise solely to active learning. However, this cohort experienced active learning and a large error burden during training, in contrast to the easy EMT cohort, which experienced active learning without a large error burden. These findings suggest that making errors during training may provide a distinct and crucial benefit for developing adaptive expertise separately from active learning. Active learning may allow generalized reflection, but making errors allows focused reflection on one’s own incomplete conceptual understanding of a topic.^7,31,40^ Our study establishes the essential groundwork for future research using EMT with other cognitive skills in medical education. Using errors during training to develop adaptive expertise may ultimately help physicians reduce errors in future practice.

### Limitations

This study has some limitations. First, we used a sample population of emergency medicine residents with a curriculum relevant to emergency medicine. Thus, the curriculum in its current format may not be directly generalizable to other specialties that interpret head CT scans, such as radiology, neurology, or neurosurgery. Second, our sample population came from 6 university-based programs and 1 county-based program, which limits generalizability to community-based programs. Third, the posttest was not directly supervised, which may limit the validity of our results. Although participants were instructed to complete the posttest individually, they may have worked together or shared answers to test questions, which may have skewed posttest scores toward being more similar, potentially reducing differences between groups. Fourth, there was a lower posttest completion rate in both EMT cohorts (64.3% and 67.6% for the difficult and easy EMT cohorts, respectively) vs the EAT control cohort (80.3%). This lower rate may indicate that the EMT cohorts had a higher proportion of more motivated residents compared with the control cohort. However, it would not explain the differences we found between the 2 EMT cohorts. Fifth, although our findings demonstrate that struggling through difficult problems develops adaptive expertise, our study cannot indicate the precise degree of difficulty required. Our future research will investigate how to refine these degrees of difficulty for a range of cognitive tasks in medical education.

## Conclusions

This randomized clinical trial shows that EMT improved emergency medicine residents’ adaptive expertise as they learned focused head CT interpretation. Making more errors during training mediated the intervention’s effect of reducing errors when residents faced novel cases on a posttest. Our findings challenge the prevailing traditional model of errorless training in medical education. This innovative method is a model for building adaptive expertise in emergency medicine and other specialties.

## Notes

Supplement 1.
Trial Protocol

Supplement 2.
eMethods.
**eTable 1.** Participant Baseline Characteristics and Proportion Within Cohorts by Posttest Completion **eTable 2.** Baseline Characteristics for All Participants Initially Enrolled **eTable 3.** Adaptive Expertise Posttest Scores Stratified by PGY Level **eTable 4.** Routine Expertise Posttest Scores Stratified by PGY Level **eTable 5.** Two-way ANOVA Assessing the Interaction Effect Between PGY Level and Learning Strategy **eTable 6.** Sensitivity Analyses for Two-Way ANOVAs of Adaptive Expertise and Routine Expertise Cases, With PGY 4 Residents Removed from the Senior Resident Factor **eFigure.** Experimental Design for the Educational Intervention.

Supplement 3.
Data Sharing Statement
