# Supplementary material for: Error Management Training and Adaptive Expertise in Learning Computed Tomography Interpretation: A Randomized Clinical Trial
Source: JAMA Netw Open. 2024 Sep 9;7(9):e2431600. doi: 10.1001/jamanetworkopen.2024.31600 (PMC11385054; doi:10.1001/jamanetworkopen.2024.31600)
Supplement: Supplement 1. — Trial Protocol [file jamanetwopen-e2431600-s001.pdf]

FAIL CT Study Protocol

Title:
The FAIL CT Study: Facilitating Adaptive Expertise in Learning Computed Tomography: A Multicenter Randomized Controlled Trial

Trial Registration:
Trial Registration: ClinicalTrials.gov Identifier: NCT05284838

Protocol Version & Updates:
1.0 – February 2, 2022, pending IRB approval and funding
1.1 – April 14, 2022: IRB approval obtained
1.2 – September 12, 2022: Received Innovation Grant from the Stanford Teaching and Mentoring Academy

Responsible Party:
Leonardo Aliaga, MD
Stanford University
Principal Investigator

INTRODUCTION

Background, Rationale, and Specific Aims:

Many medical errors are preventable[1] ; we can reduce those preventable errors by training physicians to develop adaptive expertise. Adaptive expertise is the ability to apply existing skills and knowledge to solve novel problems and manage uncommon situations.[2] This type of expertise can help physicians reduce medical errors when they manage novel clinical cases not previously encountered during training.[3] However, residency curricula often fail to emphasize and assess this core learning outcome. One missed opportunity for improving adaptive expertise is Error Management Training (EMT), a teaching method that capitalizes on the value of making mistakes when learning. Making errors while learning in non-clinical environments such as simulation improves transfer of skills to new contexts, thus reducing

errors when physician confront novel clinical scenarios.[4] Although EMT has been shown to improve adaptive expertise specifically in procedural skills, its impact on cognitive skills in medical training remains underexplored.[5–7] EMT promises to improve patient care by accelerating the development of physicians' adaptive expertise. However, to effectively incorporate EMT into residency training, we need further evidence for its efficacy in developing cognitive skills in graduate medical education and a model for modulating its components in a residency curriculum.

Our long-term goal is to create a body of evidence supporting the use of EMT in residency training to develop adaptive expertise. **The main objective of this study is to determine whether EMT improves adaptive expertise for a cognitive skill in medical education, using head computed tomography (CT) interpretation as a model.** This cognitive skill provides an ideal model for investigating EMT since we can precisely measure performance when comparing learning strategies. Our central hypothesis is that EMT, compared to error avoidance training (EAT), will improve adaptive expertise when used to teach head CT interpretation to emergency medicine residents. To test this hypothesis, we will conduct a three-arm randomized controlled trial (RCT) comparing two EMT and one EAT learning strategies. Our pilot RCT on head CT curriculum design and assessment provides the feasibility data and educational material for this study.[8] Prior simulation studies used EMT to improve transfer and adaptive expertise when teaching ultrasound, central venous catheter placement, and lower extremity fasciotomy.[5–7] Showing that EMT produces a similar outcome when used to teach the cognitive skill of head CT interpretation will provide the foundational evidence for expanding our use of EMT in residency training. Our specific aims are:

Aim 1. To determine whether EMT improves adaptive expertise with cognitive skills, as compared to EAT. We will conduct a multi-center RCT across 7-10 geographically diverse emergency medicine residency programs. We will deliver EMT and EAT head CT educational interventions virtually using a web-based radiology simulation platform and assess diagnostic accuracy using a validated head CT interpretation post-test.

Aim 2. To ascertain how the number of learner errors mediates the effect of EMT on skills transfer. We will modulate the experience of making errors between two EMT cohorts in the RCT and carry out a mediation analysis, which will elucidate the importance of making errors to achieve transfer. We hypothesize that the number of errors during training will positively mediate improvement in adaptive expertise (transfer of skills to new cases).

Aim 3. To determine whether prior residency training, as defined by higher post-graduate year (PGY) level, leads to higher adaptive expertise improvement through EMT. We will stratify post-test scores by PGY level and compare the interaction effect of the two factors: cohort and PGY level.

Using errors during training to develop adaptive expertise can ultimately help physicians reduce errors during unsupervised practice. Our work aims to provide a tool that residency programs can use to capture this benefit. This study will establish the groundwork for future experiments to determine how we can apply EMT to other cognitive skills in medical education. Producing evidence for using EMT and optimizing its implementation enables us to transform our curricula so that we can use adaptive expertise to improve patient care.

METHODS

Trial design:

A three-arm, parallel-group, blinded randomized controlled trial comparing two EMT learning strategies (EMT-1 and EMT-2) and one EAT learning strategy.

Study setting:

We have recruited 14 emergency medicine residency programs to be potential sites: Stanford University, Highland Hospital, University of California San Francisco, University of California Davis, University of California Los Angeles, University of Texas Health Science Center at Houston, University of Oklahoma Tulsa, University of Wisconsin, University of Chicago, University of Michigan, Vanderbilt, University of Alabama, The Ohio State University, and Brown University.

Participants:

Emergency medicine residents PGY 1-4 at the included programs will be eligible and invited to participate. A subset of emergency medicine residents will be part of a pilot phase of the study to collect validity evidence for the curriculum and post-test; those residents will be excluded from the study.

Study Procedure:

All participants will complete an online head CT curriculum focused on identifying intracranial hemorrhage and increased intracranial pressure, followed by a post-test. The curriculum and post-test will be completed during residents' regularly scheduled virtual didactic conference time.

Interventions:

There will be two intervention cohorts and one control cohort. Each cohort will receive the same head CT teaching cases and written educational content, however, the learning strategy used to present the content will differ between cohorts.

Teaching cases will be hosted on Pacsbin (pacsbin.com), a web-based radiology picture archiving and communication system (PACS). Pacsbin is a HIPAA compliant online platform for storing and sharing radiology cases. Pacsbin allows learners to scroll through CTs and adjust brightness/contrast as they would on their institution's radiology PACS. Scrolling through head CTs reproduces the cognitive activity used to identify critical findings in clinical practice. We previously collected head CT cases from patient encounters in the emergency department (ED), anonymized them, and uploaded them to Pacsbin. These cases represent common diagnoses encountered in the ED (e.g., intracranial hemorrhage, increased intracranial pressure) and include the critical findings that would change management.

For each teaching case, residents in the EMT-1 cohort will first have to scroll through the head CT with no guidance on how to identify the critical findings. Then they answer a brief set of questions about that CT. Residents will immediately see which of their answers are correct and incorrect. These are difficult questions that residents are likely to answer incorrectly, thus making them aware that they made errors. After answering the questions, they go to an explanations page on Pacsbin. This explanations page uses the same head CT case they just scrolled through. The written teaching points walk them through the scan and show them how to identify the critical findings. The EMT-2 cohort is identical to the EMT-1 cohort with the exception that the EMT-2 cohort encounters easy questions that they are likely to answer correctly, thus preventing them from having the overt experience of making errors. The EAT cohort will receive instructions on how to read a head CT and will scroll through the CT as directed by the on-screen instructions. They navigate directly to the explanations page for each case, thus preventing them from having a period of free exploration while scrolling through the CT without guidance and preventing them from

having the overt experience of making errors. We will pilot this curriculum to collect validity evidence for the difficult and easy questions embedded within the curriculum.

Outcomes:

Our primary outcome is adaptive expertise, as measured by learners' scores on a head CT interpretation test using cases that contain findings related to but not addressed during the teaching session, thus assessing their ability to transfer their skills. There will be three secondary outcomes. First, we will assess immediate post-session proficiency (routine expertise), as measured by learners' scores on a head CT interpretation using cases that specifically assess the direct learning objectives from the teaching session. Second, we will assess the mediation effect of errors made during training. We will measure the number of errors made during the training phase by the EMT-1 and EMT-2 cohorts, and then conduct a mediation analysis using the number of errors made during training as the mediator variable. Third, we will assess the effect of prior residency training on both adaptive and routine expertise, by first stratifying post-test scores by PGY level and then comparing interaction effects between cohort and PGY level. We adapted a head CT interpretation test from a prior study and will collect validity evidence for using the test in this study. The prior study collected content and response process validity evidence.[8] We will conduct a pilot phase to collect additional response process validity evidence and conduct item analysis using discrimination index and item difficulty.

Sample size:

A sample size of 46 subjects per cohort will have 80% power at the 0.05 significance level to detect a difference of 6% (medium effect size) between EAT and either EMT cohort (in a conservative model with no difference between the two EMT cohorts). We used the standard deviation from a validated head CT interpretation test from a prior study.

Recruitment:

Emergency medicine residents PGY 1-4 in included programs will be invited by email to participate in the study. They will receive a consent form describing the study and their involvement. The sites leads at each residency program will also announce the study.

Allocation:

Each residency progam will undergo its own randomization process (to account for program-level confounders). Residents who consent to participate will be stratified by PGY and then

block randomized (1:1:1) to one of the three cohorts. Randomization blocks (block size of 3) were generated using Research Randomizer (www.randomizer.org).

Blinding:

Participants are blinded to the study hypothesis. Participants will be assigned a unique, random study identifier which will be used to deidentify their post-test scores.

Data collection and management:

Post-test scores will be stored on REDCap and accessed via a password-protected computer, using the Stanford Whole Disk Encryption.

Statistical Methods:

We will compare post-test scores on the adaptive and routine expertise cases using one-way analysis of variance (ANOVA), with planned pairwise comparisons between the three cohorts. We will compare the number of errors made on the training cases by the EMT-1 and EMT-2 cohorts using a two-tailed independent t-test. We will then perform a mediation analysis using Hayes PROCESS macro for SPSS, with learning strategy as the independent variable, number of errors during training as the mediator variable, and post-test score on adaptive expertise cases as the outcome variable. We will assess the effect of PGY level on post-test scores using two-way ANOVAs (factors were cohort and PGY level). We will calculate effect sizes using η^2 and Cohen's d, where applicable. We will perform all statistical analyses on SPSS version 28 (IBM).

Ethics:

Participants will receive a consent form via automated email from REDCap at least two weeks before the intervention, describing the study and their involvement. Their written consent will be stored on REDCap. Site leads will not have access to these consent forms. The participants will understand that their decision to participate will be kept anonymous from their residency leadership and that their decision to participate will not affect their evaluation as a resident.

Participation in this study will pose minimal risks to subjects. Participants may feel some cognitive discomfort completing the head CT instructional session since the training will be dissimilar from the lectures they are accustomed to receiving. They may also feel some

cognitive discomfort while completing the post-test. Residency program leadership will not have access to their residents' individual post-test scores.

This study has been approved by the Stanford Institutional Review Board on 4/14/2022, protocol # 64099.

REFERENCES

1. *To Err Is Human: Building a Safer Health System*. National Academies Press; 2000. Accessed January 26, 2022. http://www.nap.edu/catalog/9728

2. Pusic MV, Santen SA, Dekhtyar M, et al. Learning to balance efficiency and innovation for optimal adaptive expertise. *Medical Teacher*. 2018;40(8):820-827.

3. Royce CS, Hayes MM, Schwartzstein RM. Teaching Critical Thinking: A Case for Instruction in Cognitive Biases to Reduce Diagnostic Errors and Improve Patient Safety. *Academic Medicine*. 2019;94(2):187-194.

4. King A, Holder MG, Ahmed RA. Errors as allies: error management training in health professions education. *BMJ Qual Saf*. 2013;22(6):516-519.

5. Dyre L, Tabor A, Ringsted C, Tolsgaard MG. Imperfect practice makes perfect: error management training improves transfer of learning. *Med Educ*. 2017;51(2):196-206.

6. Gardner AK, Abdelfattah K, Wiersch J, Ahmed RA, Willis RE. Embracing Errors in Simulation-Based Training: The Effect of Error Training on Retention and Transfer of Central Venous Catheter Skills. *Journal of Surgical Education*. 2015;72(6):e158-e162.

7. Franklin BR, Dyke C, Durning SJ, et al. Piloting the FIRE: A Novel Error Management Training Simulation Curriculum for Fasciotomy Instruction. *Journal of Surgical Education*. 2021;78(2):655-664.

8. Aliaga L, Clarke S. Rethinking Radiology: An Active Learning Curriculum for Head Computed Tomography Interpretation. *WestJEM*. 2022;23(1):47-51.

STANFORD UNIVERSITY

Stanford, CA 94305 [Mail Code 5579]

David D Oakes, M.D. (650) 723-5215
CHAIR, PANEL ON MEDICAL HUMAN SUBJECTS (650) 725-8013

Certification of Human Subjects Approvals

Date: April 14, 2022

To: Leonardo Aliaga, MD, Emergency Medicine
Dr Michael Gisondi MD, Holly Caretta-Weyer, Stefanie Syer

From: David D Oakes, M.D., Administrative Panel on Human Subjects in Medical Research

eProtocol The FAIL CT Study: Facilitating Adaptive Expertise in Learning Computed Tomography, a Multi-center Randomized Controlled Trial

eProtocol #: 64099 **IRB 8 (Registration** 6208)

Overall risk level: Minimal risk

The IRB approved human subjects involvement in your research project on 04/14/2022. **'Prior to subject recruitment and enrollment, if this is: a Cancer-related study, you must obtain Cancer Center Scientific Review Committee (SRC) approval;** a CTRU study, you must obtain CTRU approval; a VA study, you must obtain VA R and D Committee approval; and **if a contract is involved, it must be signed.** '

This protocol has been approved under the Extended Approval Process and **approval does not expire**. Proposed changes to approved research must still be reviewed and approved prospectively by the IRB. No changes may be initiated without prior approval by the IRB, except where necessary to eliminate apparent immediate hazards to subjects. (Any such exceptions must be reported to the IRB within 10 working days.) Unanticipated problems involving risks to participants or others and other events or information, as defined and listed in the Report Form, must be submitted promptly to the IRB. (See Events and Information that Require Prompt Reporting to the IRB at http://humansubjects.stanford.edu.) It is your responsibility to report the completion of the protocol to the IRB within 30 days.

Please remember that all data, including all signed consent form documents, must be retained for a minimum of three years past the completion of this research. Additional requirements may be imposed by your funding agency, your department, HIPAA, or other entities. (See Policy 1.9 on Retention of and Access to Research Data at http://doresearch.stanford.edu/policies/research-policy-handbook)

This institution is in compliance with requirements for protection of human subjects, including 45 CFR 46, 21 CFR 50 and 56, and 38 CFR 16.

Includes: The protocol was approved with the understanding that data will not be shared between sites until copies of the collaborating sites IRB approval letters have been provided to the Stanford IRB.

David D Oakes, M.D., Chair

Approval Period: 04/14/2022 - (Does Not Expire)

Review Type: EXPEDITED - NEW

Funding: Emergency Medicine Foundation, Council of Residency Directors in Emergency Medicine, SPO: pending

Expedited Under Category: 7

Assurance #: FWA00000935 (SU), FWA00000934 (SHC)
