# Supplementary material for: Error Management Training and Adaptive Expertise in Learning Computed Tomography Interpretation: A Randomized Clinical Trial
Source: JAMA Netw Open. 2024 Sep 9;7(9):e2431600. doi: 10.1001/jamanetworkopen.2024.31600 (PMC11385054; doi:10.1001/jamanetworkopen.2024.31600)
Supplement: Supplement 2. — eMethods. eTable 1. Participant Baseline Characteristics and Proportion Within Cohorts by Posttest Completion eTable 2. Baseline Characteristics for All Participants Initially Enrolled eTable 3. Adaptive Expertise Posttest Scores Stratified by PGY Level eTable 4. Routine Expertise Posttest Scores Stratified by PGY Level eTable 5. Two-way ANOVA Assessing the Interaction Effect Between PGY Level and Learning Strategy eTable 6. Sensitivity Analyses for Two-Way ANOVAs of Adaptive Expertise and Routine Expertise Cases, With PGY 4 Residents Removed from the Senior Resident Factor eFigure. Experimental Design for the Educational Intervention. [file jamanetwopen-e2431600-s002.pdf]

# Supplemental Online Content

Aliaga L, Bavolek RA, Cooper B, et al. Error management training and adaptive expertise in learning computed tomography interpretation: a randomized clinical trial. *JAMA Netw Open*. 2024;7(9):e2431600. doi:10.1001/jamanetworkopen.2024.31600

**eMethods**

**eTable 1.** Participant Baseline Characteristics and Proportion Within Cohorts by Posttest Completion

**eTable 2.** Baseline Characteristics for All Participants Initially Enrolled

**eTable 3.** Adaptive Expertise Posttest Scores Stratified by PGY Level

**eTable 4.** Routine Expertise Posttest Scores Stratified by PGY Level

**eTable 5.** Two-way ANOVA Assessing the Interaction Effect Between PGY Level and Learning Strategy

**eTable 6.** Sensitivity Analyses for Two-Way ANOVAs of Adaptive Expertise and Routine Expertise Cases, With PGY 4 Residents Removed from the Senior Resident Factor

**eFigure.** Experimental Design for the Educational Intervention.

This supplemental material has been provided by the authors to give readers additional information about their work.

© 2024 Aliaga L et al. *JAMA Network Open*.

eMethods.

Training modules were delivered on websites, one for each cohort:

Difficult-EMT: https://www.failct-1.org

Easy-EMT: https://www.tmecemt.org

EAT control: https://www.aectcea-1.org

Link to head CT interpretation post-test: FAIL CT Study Post-test

© 2024 Aliaga L et al. *JAMA Network Open*.

**eTable 1.** Participant Baseline Characteristics and Proportion Within Cohorts by Posttest Completion

| | Participants, No. (%) | | | |
|---|---|---|---|---|
| Characteristic | Overall (n=212) | Completed the post-test (n=150) | Did NOT complete the post-test (n=62) | *P* value [a] |
| Age, mean (SD), y | 28.8 (2.0) | 28.9 (2.1) | 28.4 (1.7) | .08 |
| Gender | | | | |
| Women | 105 (49.5) | 74 (49.3) | 31 (50.0) | .93 |
| Men | 107 (50.5) | 76 (50.7) | 31 (50.0) | |
| Post-graduate year | | | | |
| 1 | 68 (32.1) | 45 (30.0) | 23 (37.1) | .74 |
| 2 | 65 (30.7) | 48 (32.0) | 17 (27.4) | |
| 3 | 62 (29.2) | 44 (29.3) | 18 (29.0) | |
| 4 | 17 (8.0) | 13 (8.7) | 4 (6.5) | |
| Institution | | | | |
| University of California Los Angeles | 40 (18.9) | 28 (18.7) | 12 (19.4) | .69 |
| Highland Hospital | 23 (10.8) | 16 (10.7) | 7 (11.3) | |
| Brown University | 30 (14.2) | 19 (12.7) | 11 (17.7) | |
| University of Chicago | 25 (11.8) | 15 (10.0) | 10 (16.1) | |
| University of Wisconsin | 24 (11.3) | 18 (12.0) | 6 (9.7) | |
| UT Health Houston | 46 (21.7) | 36 (24.0) | 10 (16.1) | |
| Vanderbilt University | 24 (11.3) | 18 (12.0) | 6 (9.7) | |
| Program length | | | | |
| 4-year programs | 93 (43.9) | 63 (42.0) | 30 (48.4) | .39 |
| 3-year programs | 119 (56.1) | 87 (58.0) | 32 (51.6) | |
| Cohort | | | | |
| Difficult-EMT | 70 (33.0) | 45 (30.0) | 25 (40.3) | .09 |
| Easy-EMT | 71 (33.5) | 48 (32.0) | 23 (37.1) | |
| EAT Control | 71 (33.5) | 57 (38.0) | 14 (22.6) | |

© 2024 Aliaga L et al. *JAMA Network Open*.

[a] Mann-Whitney U test; Pearson's Chi-squared test

© 2024 Aliaga L et al. *JAMA Network Open*.

**eTable 2.** Baseline Characteristics for All Participants Initially Enrolled

| | Participants, No. (%) | | | | |
|---|---|---|---|---|---|
| Characteristic | Overall (n=212) | Difficult-EMT (n=70) | Easy-EMT (n=71) | EAT Control (n=71) | *P* value [a] |
| Age, mean (SD), y | 28.8 (2.0) | 28.6 (2.1) | 28.9 (2.3) | 28.8 (1.6) | .22 |
| Gender | | | | | |
| Women | 105 (49.5) | 32 (45.7) | 36 (50.7) | 37 (52.1) | .73 |
| Men | 107 (50.5) | 38 (54.3) | 35 (49.3) | 34 (47.9) | |
| Post-graduate year | | | | | |
| 1 | 68 (32.1) | 24 (34.3) | 22 (31.0) | 22 (31.0) | .72 |
| 2 | 65 (30.7) | 22 (31.4) | 22 (31.0) | 21 (29.6) | |
| 3 | 62 (29.2) | 21 (30.0) | 22 (31.0) | 19 (26.8) | |
| 4 | 17 (8.0) | 3 (4.3) | 5 (7.0) | 9 (12.7) | |
| Institution | | | | | |
| University of California Los Angeles | 40 (18.9) | 13 (18.6) | 13 (18.3) | 14 (19.7) | 1.0 |
| Highland Hospital | 23 (10.8) | 8 (11.4) | 8 (11.3) | 7 (9.9) | |
| Brown University | 30 (14.2) | 10 (14.3) | 10 (14.1) | 10 (14.1) | |
| University of Chicago | 25 (11.8) | 8 (11.4) | 9 (12.7) | 8 (11.3) | |
| University of Wisconsin | 24 (11.3) | 8 (11.4) | 8 (11.3) | 8 (11.3) | |
| UT Health Houston | 46 (21.7) | 15 (21.4) | 15 (21.1) | 16 (22.5) | |
| Vanderbilt University | 24 (11.3) | 8 (11.4) | 8 (11.3) | 8 (11.3) | |
| Program length | | | | | |
| 4-year programs | 93 (43.9) | 31 (44.3) | 31 (43.7) | 31 (43.7) | .99 |
| 3-year programs | 119 (56.1) | 39 (55.7 | 40 (56.3) | 40 (56.3) | |

[a] Kruskal-Wallis test; Pearson's Chi-squared test
Abbreviations: EMT, Error Management Training; EAT, Error Avoidance Training.

© 2024 Aliaga L et al. *JAMA Network Open*.

**eTable 3.** Adaptive Expertise Posttest Scores Stratified by PGY Level

| | Adaptive expertise post-test score: Mean (95% CI) | | | ANOVA | |
|---|---|---|---|---|---|
| | Difficult-EMT | Easy-EMT | EAT Control | *P* value | $\eta^2$ |
| PGY 1 | 70.5 (64.1 to 76.8) | 40.2 (32.7 to 47.7) | 35.7 (26.2 to 45.3) | <.001 | 0.56 |
| PGY 2 | 59.2 (52.0 to 66.3) | 44.8 (31.5 to 58.1) | 37.6 (29.6 to 45.6) | .006 | 0.20 |
| PGY 3&4 | 52.7 (44.5 to 60.9) | 50.4 (42.2 to 58.7) | 46.4 (38.0 to 54.8) | .53 | NA |

Abbreviations: EMT, Error Management Training; EAT, Error Avoidance Training; PGY, post-graduate year

© 2024 Aliaga L et al. *JAMA Network Open*.

**eTable 4.** Routine Expertise Posttest Scores Stratified by PGY Level

| | Routine expertise post-test score: Mean (95% CI) | | | ANOVA | |
|---|---|---|---|---|---|
| | Difficult-EMT | Easy-EMT | EAT Control | *P* value | $\eta^2$ |
| PGY 1 | 74.9 (70.9 to 78.9) | 66.4 (58.0 to 74.8) | 69.7 (62.7 to 76.7) | .17 | NA |
| PGY 2 | 73.3 (65.7 to 80.1) | 72.6 (63.9 to 81.2) | 71.7 (65.7 to 77.7) | .94 | NA |
| PGY 3&4 | 76.8 (71.3 to 82.2) | 81.2 (76.5 to 86.0) | 79.2 (75.7 to 82.6) | .38 | NA |

Abbreviations: EMT, Error Management Training; EAT, Error Avoidance Training; PGY, post-graduate year

© 2024 Aliaga L et al. *JAMA Network Open*.

**eTable 5.** Two-way ANOVA Assessing the Interaction Effect Between PGY Level and Learning Strategy

| | Adaptive expertise cases | | Routine expertise cases | |
|---|---|---|---|---|
| Factor | *P* value | $\eta^2$ | *P* value | $\eta^2$ |
| Cohort | <.001 | 0.22 | .77 | NA |
| PGY level | .73 | NA | <.001 | 0.10 |
| Cohort-PGY interaction | .006 | 0.10 | .29 | NA |

Abbreviations: PGY, post-graduate year

© 2024 Aliaga L et al. *JAMA Network Open*.

**eTable 6.** Sensitivity Analyses for Two-Way ANOVAs of Adaptive Expertise and Routine Expertise Cases, With PGY 4 Residents Removed from the Senior Resident Factor

| | Adaptive expertise cases | | Routine expertise cases | |
|---|---|---|---|---|
| Factor | *P* value | $\eta^2$ | *P* value | $\eta^2$ |
| Cohort | <.001 | 0.24 | .81 | NA |
| PGY level | .77 | NA | .008 | 0.07 |
| Cohort-PGY interaction | .01 | 0.10 | .34 | NA |

Abbreviations: PGY, post-graduate year

© 2024 Aliaga L et al. *JAMA Network Open*.

**eFigure.** Experimental Design for the Educational Intervention

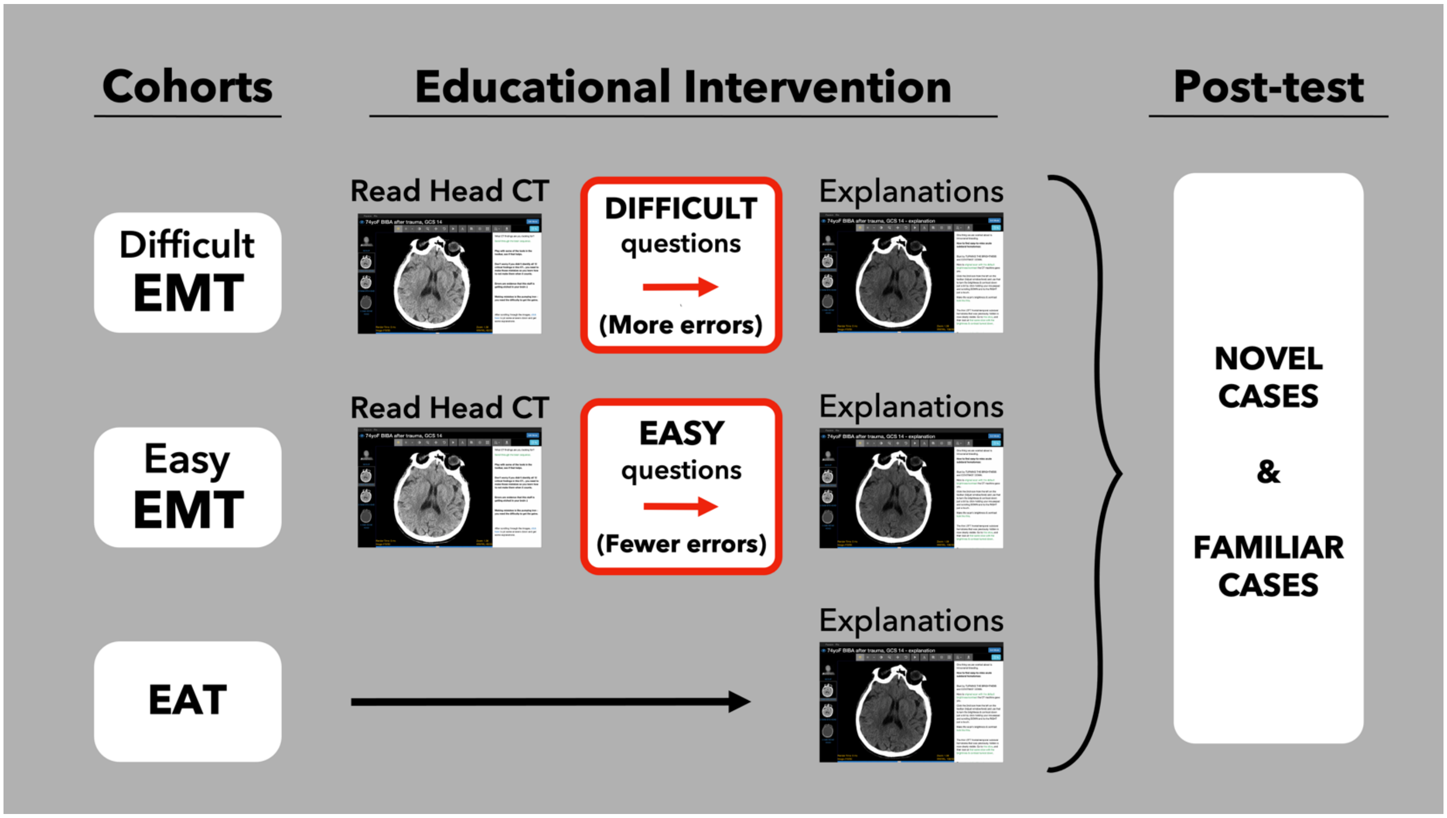


Abbreviations: EMT, Error Management Training; EAT, Error Avoidance Training; CT, computed tomography

© 2024 Aliaga L et al. *JAMA Network Open*.
