# Supplementary material for: Error Management Training and Adaptive Expertise in Learning Computed Tomography Interpretation: A Randomized Clinical Trial
Source: JAMA Netw Open. 2024 Sep 9;7(9):e2431600. doi: 10.1001/jamanetworkopen.2024.31600 (PMC11385054; doi:10.1001/jamanetworkopen.2024.31600)
Supplement: Supplement 3. — Data Sharing Statement [file jamanetwopen-e2431600-s003.pdf]

Data Sharing Statement

Aliaga. Error Management Training and Adaptive Expertise in Learning Computed Tomography Interpretation. *JAMA Netw Open*. Published September 09, 2024. doi:10.1001/jamanetworkopen.2024.31600

Data
Data available: Yes
Data types: Deidentified participant data
How to access data: Upon request from aliagal@stanford.edu.
When available: With publication

Supporting Documents
Document types: None

Additional Information
Who can access the data: Researchers whose proposed use of the data has been approved.
Types of analyses: Any purpose.
Mechanisms of data availability: With a signed data access agreement.
